# Transposable element polymorphisms recapitulate human evolution

**DOI:** 10.1186/s13100-015-0052-6

**Published:** 2015-11-16

**Authors:** Lavanya Rishishwar, Carlos E. Tellez Villa, I. King Jordan

**Affiliations:** School of Biology, Georgia Institute of Technology, 310 Ferst Drive, Atlanta, GA 30332-0230 USA; PanAmerican Bioinformatics Institute, Cali, Valle del Cauca Colombia; BIOS Centro de Bioinformática y Biología Computacional, Manizales, Caldas Colombia; Escuela de Ingeniería de Sistemas y Computación, Universidad del Valle, Santiago de Cali, Colombia

**Keywords:** Transposable elements, Polymorphism, Population genetics, Human ancestry, Admixture, Ancestry informative markers, Phylogenetics, Alu, L1, SVA

## Abstract

**Background:**

The human genome contains several active families of transposable elements (TE): Alu, L1 and SVA. Germline transposition of these elements can lead to polymorphic TE (polyTE) loci that differ between individuals with respect to the presence/absence of TE insertions. Limited sets of such polyTE loci have proven to be useful as markers of ancestry in human population genetic studies, but until this time it has not been possible to analyze the full genomic complement of TE polymorphisms in this way.

**Results:**

For the first time here, we have performed a human population genetic analysis based on a genome-wide polyTE data set consisting of 16,192 loci genotyped in 2,504 individuals across 26 human populations. PolyTEs are found at very low frequencies, > 93 % of loci show < 5 % allele frequency, consistent with the deleteriousness of TE insertions. Nevertheless, polyTEs do show substantial geographic differentiation, with numerous group-specific polymorphic insertions. African populations have the highest numbers of polyTEs and show the highest levels of polyTE genetic diversity; Alu is the most numerous and the most diverse polyTE family. PolyTE genotypes were used to compute allele sharing distances between individuals and to relate them within and between human populations. Populations and continental groups show high coherence based on individuals’ polyTE genotypes, and human evolutionary relationships revealed by these genotypes are consistent with those seen for SNP-based genetic distances. The patterns of genetic diversity encoded by TE polymorphisms recapitulate broad patterns of human evolution and migration over the last 60–100,000 years. The utility of polyTEs as ancestry informative markers is further underscored by their ability to accurately predict both ancestry and admixture at the continental level. A genome-wide list of polyTE loci, along with their population group-specific allele frequencies and F_ST_ values, is provided as a resource for investigators who wish to develop panels of TE-based ancestry markers.

**Conclusions:**

The genetic diversity represented by TE polymorphisms reflects known patterns of human evolution, and ensembles of polyTE loci are suitable for both ancestry and admixture analyses. The patterns of polyTE allelic diversity suggest the possibility that there may be a connection between TE-based genetic divergence and population-specific phenotypic differences.

**Graphical Abstract:**
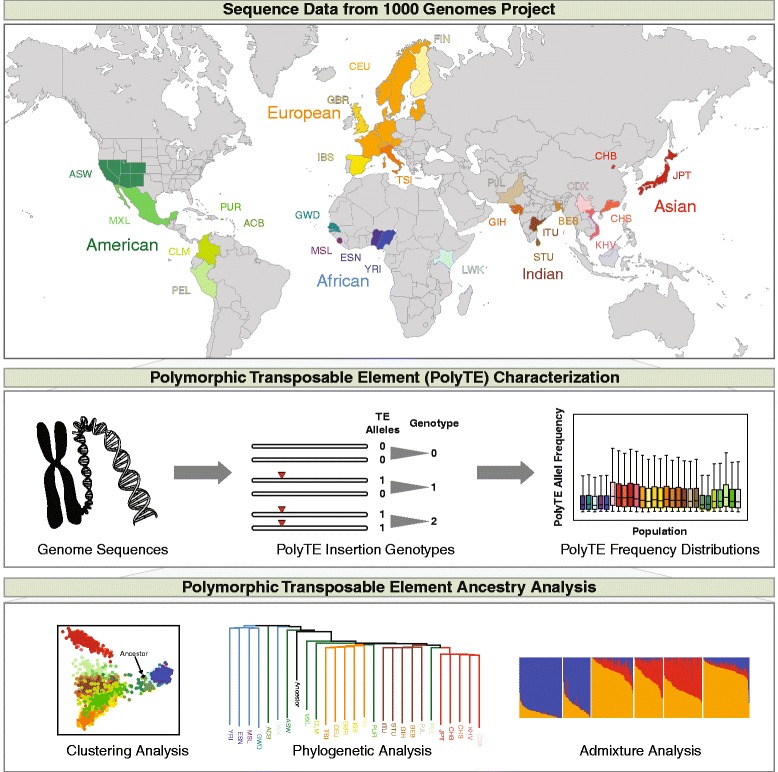
ᅟ

**Electronic supplementary material:**

The online version of this article (doi:10.1186/s13100-015-0052-6) contains supplementary material, which is available to authorized users.

## Background

Much of the human genome sequence, anywhere from ~50 to 70 % depending on estimates [1, 2], is derived from transposable elements (TE). The vast majority of TE-derived sequences in the genome are remnants of ancient insertion events, which are no longer capable of transposition. Nevertheless, there remain a few families of actively transposing human TEs [3]; the active families of human TEs include Alu [4, 5], L1 [6, 7] and SVA [8, 9] elements. Alu elements are 7SL RNA-derived short interspersed nuclear elements (SINEs) [10, 11], L1s are a family of long interspersed nuclear elements (LINEs) [12, 13], and SVA elements are composite TEs that are made up of human endogenous retrovirus sequence, simple sequence repeats and Alu sequence [14, 15]. All three of these active families of human TEs are retrotransposons that transpose via reverse transcription of an RNA intermediate. L1s are autonomous retrotransposons that encode the enzymatic machinery necessary to catalyze their own retrotransposition [16], whereas Alu and SVA elements are transposed in *trans* by the L1 machinery [17, 18].

If members of these active TE families transpose in the germline, they can create novel insertions that are capable of being inherited, thereby generating human-specific polymorphisms. Such polymorphic TE (polyTE) insertion sites have been shown to be valuable genetic markers for studies of human ancestry and evolution. PolyTEs provide a number of advantages for such population genetic studies [3, 19]. First, the presence of a polyTE insertion site shared by two or more individuals nearly always represents identity by descent [19, 20]. This is because there are so many possible insertion sites genome-wide, and transposition rates are so low, that the probability of independent insertion at the same site in two individuals is negligible. Second, since newly inserted TEs rarely undergo deletion they are highly stable polymorphisms. These two characteristics underscore the fact that polyTE markers are completely free of homoplasies, i.e. identical states that do not represent shared ancestry, which are far more common for single nucleotide polymorphisms (SNPs). Another useful feature of polyTEs for population genetic studies is the fact that the ancestral state of polyTE loci is known to be absence of the insertion [21, 22]. Finally, polyTEs are practically useful markers since they can be rapidly and accurately typed via PCR-based assays.

A number of previous studies have leveraged TE polymorphisms for the analysis of human ancestry and evolution [3, 18, 19, 21–27]. Most of these studies have focused on Alu elements; there have been far fewer human population genetic studies using L1 markers and to our knowledge no such studies using polymorphic SVA elements. Alus are particularly advantageous for these types of studies because their small size allows them to be readily PCR amplified; furthermore, both the presence and absence of Alu insertions can yield amplification products from a single PCR. Ancestry studies that use TE polymorphisms have relied on a number of selection criteria in order to try and define the most useful polyTE loci for human population differentiation. For instance, polyTE loci have often been identified via literature surveys of specific gene mutations caused by TE insertions. Analysis of the human genome sequence has also been used to identify intact members of the youngest (i.e. recently active) subfamilies of Alus and L1s in order to try and predict potentially mobile sequences. Once potential polyTE marker loci are chosen using these methods, they need to be empirically evaluated with respect to their levels of polymorphism within and between populations. These approaches, while somewhat *ad hoc* and laborious, have in fact proven to be useful for the identification of polyTE loci that serve as ancestry informative markers (AIMs).

The most recent data release from the 1000 Genome Project (Phase3 November 2014) includes, for the first time, a comprehensive genome-wide data set of polyTE sites. There are a total of 16,192 such polyTE loci reported for 2,504 individuals across 26 human populations. These newly available data provide an unprecedented level of depth and resolution for polyTE-based studies of human ancestry and evolution. With these data, it is now possible to evaluate the relationship between TE polymorphism and human evolution in a systematic and unbiased way. In addition, individual polyTE loci genome-wide can be evaluated with respect to their utility as AIMs as well as their applicability to ancestry studies for specific population groups. Such an analysis could provide a useful resource for investigators interested in conducting their own targeted studies on specific populations. With such a comprehensive, genome-wide polyTE data set, it is also possible to evaluate the marker utility of previously under-utilized L1 and SVA sequences. For this study, we have conducted a genome-wide population genetic analysis of human TE polymorphisms in order to address precisely these kinds of issues. This work represents the most comprehensive study of human polyTEs to date.

## Results

### Human population genomics of polyTEs

There are three families of polymorphic transposable elements (polyTEs) that show variation in presence/absence patterns at individual insertion sites across human genome sequences; these are Alu (SINE), L1 (LINE) and chimeric SVA elements. The Phase3 data release (November 2014) of the 1000 Genomes Project provides the most complete catalog of human transposable element insertion site polymorphisms available to date. Presence/absence genotypes for these human polyTEs are available for 2,504 individuals from 26 human populations across 16,192 genomic sites.

**Fig. 1:**
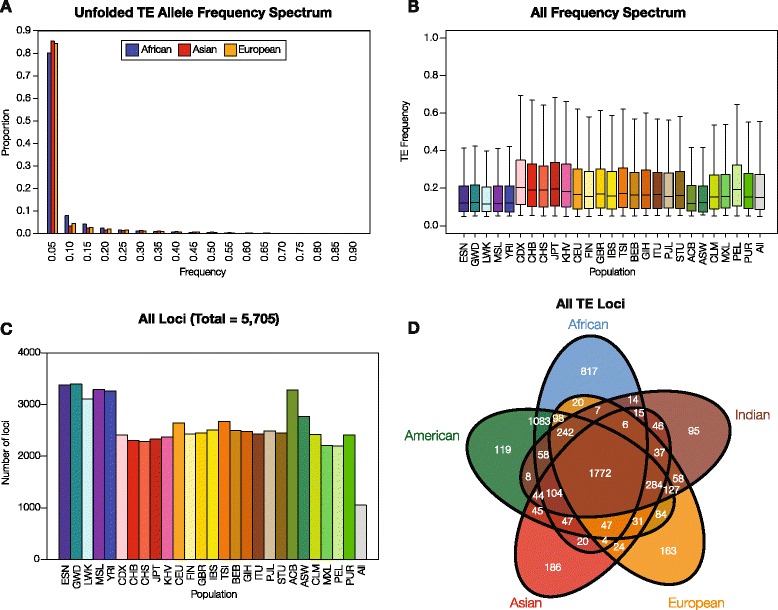
Distribution of polymorphic transposable element (polyTE) loci among human populations. Populations are organized into five continental groups (see Table 1): African (blue), Asian (red), European (gold), Indian (brown) and American (green). **a**. Unfolded polyTE allele frequency spectrum for the three ancestral (non-admixed) continental groups: African, Asian and European. **b**. Boxplot polyTE allele frequency distributions for TE insertions present at >5 % frequency within individual populations. **c**. Numbers of polyTE loci at >5 % frequency that are shared or exclusive among continental groups. **d**. Numbers of polyTE loci at >5 % frequency among the different populations

We characterized the frequencies and distributions of human polyTEs for the 26 populations organized into 5 continental groups: African, Asian, European, Indian and American (Table 1). The vast majority of human polyTEs are found at low frequencies within and between human populations; 15,141 (93.5 %) of polyTE loci show < 5 % overall allele frequencies (Fig. 1a). Nevertheless, there is substantial variability of individual polyTE allele frequencies among populations from different continental groups (Fig. 1b). Accordingly, there are higher numbers of polyTEs with continental group-specific allele frequencies > 5 % (Fig. 1c), and numerous individual polyTE loci are exclusively present within a single continental group (Fig. 1d). On average, ~25 % of individual polyTE loci are exclusive to a specific continental group. These results are consistent with the possibility that polyTE genotypes may serve as useful markers of genomic ancestry. Results of the same analyses are shown for individual polyTEs families in Additional file 1: Figure S1. Alu is by far the most abundant family of polyTEs followed by L1 and SVA. All three polyTE families show similar levels of continental group-specific insertions.

**Table 1:**
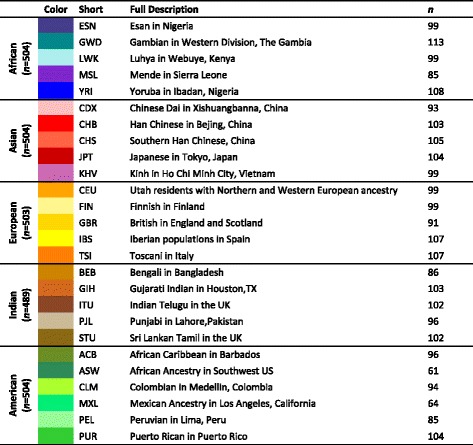
Human populations analyzed in this study

Populations are organized into five continental groups, and the number of individuals in each population is shown. The same population-specific color codes are used throughout the manuscript

**Fig. 2:**
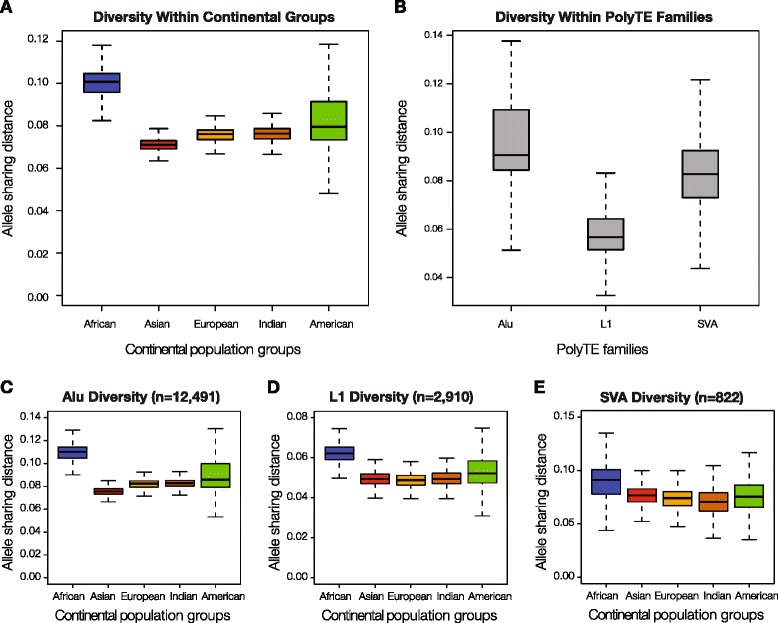
PolyTE genetic diversity levels. **a**. Distributions of overall polyTE genotype-based allele sharing distances are shown for the five continental groups (see Table 1): African (blue), Asian (red), European (gold), Indian (brown) and American (green). **b**. Distributions of polyTE genotype-based allele sharing distances are shown separately for Alu, L1 and SVA. **c**-**e**. TE family-specific distributions of polyTE genotype-based allele sharing distances are shown for separately Alu, L1 and SVA

PolyTE genotypes were analyzed in order to evaluate the polyTE genetic diversity levels for different continental groups and for different TE families. To do this, presence/absence patterns at all polyTE loci were used to genotype individual human genomes and pairwise allele sharing distances between individuals were computed based on these polyTE genotypes (see Methods). African populations have the highest levels of polyTE genetic diversity and Asian populations show the lowest diversity (Fig. 2a). These data are similar to what has been shown in previous studies of polyTEs [27] and for SNP-based genetic diversity [28]. All of the differences in median genetic diversity levels between pairs of population groups are highly statistically significant (0 ≤ *P* ≤ 8.5 x 10^−56^ Wilcoxon ranked sum test). African populations also have the highest levels of variation in polyTE genetic diversity for any of the non-admixed groups, consistent with human origins in Africa and the bottleneck experienced by other population groups during their migrations out of Africa [29, 30]. The overall effect of recent admixture in the Americas is revealed by the broad distribution of polyTE genetic diversity among the American populations, and African admixture among these same populations probably accounts for the fact that this group has the second highest level of median diversity seen for all continental groups (Fig. 2a). For polyTE families, Alu has the highest diversity followed by SVA and L1 (Fig. 2b). The relative levels of continental group polyTE genetic diversity are the same for all three families of polyTEs (Fig. 2c–d).

### Human evolutionary relationships based on polyTEs

**Fig. 3:**
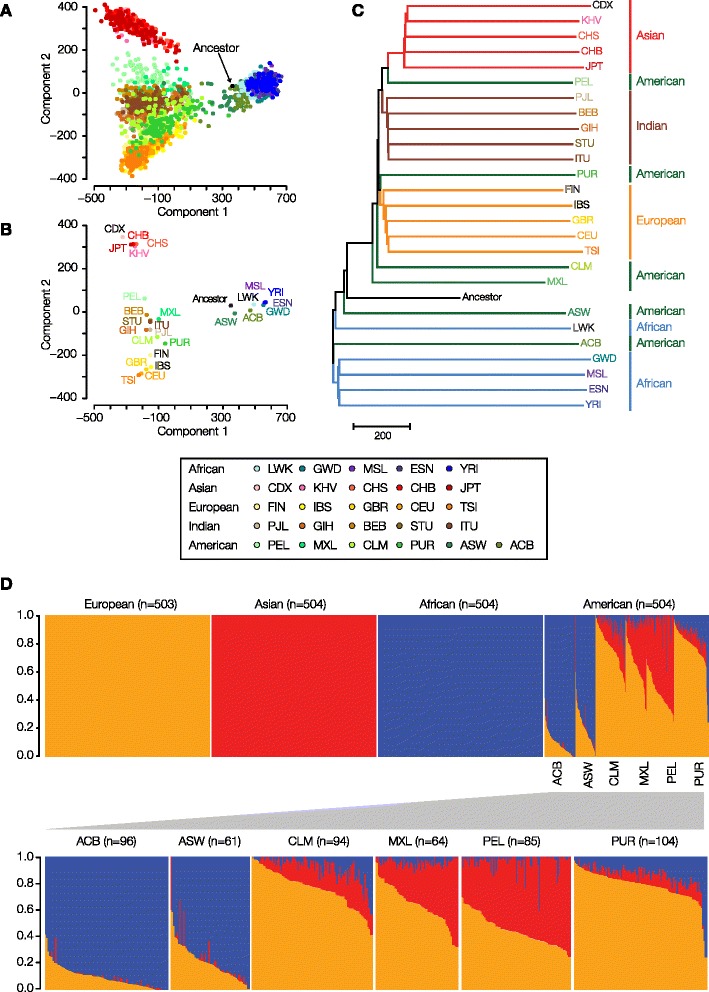
Evolutionary relationships among human populations based on polyTE genotypes. Populations are color coded as shown in the figure legend. **a**. Multi-dimensional scaling (MDS) plot showing polyTE genotype-based distances among 2,504 individuals from 26 human populations. **b**. The same polyTE genotype MDS plot showing population average distances. **c**. Phylogenetic tree based on average polyTE allele sharing distances between human populations. **d**. polyTE genotype-based continental ancestry contribution fractions for individuals from non-admixed ancestral (European, Asian and African) and admixed (American) human populations. An expanded view of the ancestry fractions is shown for the admixed American populations

The distributions of polyTE genotypes among individuals were analyzed in an effort to reconstruct the evolutionary relationships among human individuals and populations. To do this, PolyTE genotype allele sharing distances were used to generate multi-dimensional scaling (MDS) plots showing the genetic relationships among all individuals (Fig. 3a) and the average genetic relationships between individual populations (Fig. 3b). Phylogenetic reconstruction was also used to show the average polyTE genotype-based relationships between populations (Fig. 3c). The evolutionary relationships revealed by this analysis are entirely consistent with previous analyses based on individual nucleotide level variation assessed via SNP-based genotypes [31], and very similar to what has previously been seen based on Alu polymorphisms [23]. African, Asian and European continental groups represent the three poles of human genomic variation with the more ancient admixed Indian group and more recent admixed American group in between. In the phylogenetic analysis, the African populations are the most basal with the European and Asian populations being derived.

One of the advantages of using TE polymorphisms for ancestry inference is that the ancestral state for any polyTE loci can be confidently taken to be the absence of an insertion [21, 22]. This property allows for the creation of a hypothetical ancestral genome characterized by the absence of insertions across all polyTE loci. When such a hypothetical ancestor is included in the polyTE-based reconstruction of human evolutionary relationships, it maps near the center of the MDS plots closer to the African populations (Fig. 3a and b), and it maps closest to the root of the phylogeny between the African and non-African lineages (Fig. 3c). These results confirm that polyTE insertions are derived allelic states.

For the most part, there is high coherence of polyTE genotypes within both individual populations and for continental groups. The only exception seen is for the admixed American continental group, which has two distinct subgroups, a Latino subgroup (PEL, MXL, CLM and PUR) with primarily European and Asian admixture and an African-American subgroup (ACB and ASW) with primarily African and European admixture (Fig. 3d). The relative admixture levels seen for these populations are consistent with previous nucleotide level SNP-based analysis [32, 33]. The apparent Asian admixture of the Latino subgroup reflects Native American ancestry owing to the fact that Native Americans are relatively recently derived from East Asian populations [34]. As there are no Native American samples in the 1000 Genomes Project Data [28, 35], the East Asian genome sequences appear as most closely related to the Latino subgroup. CLM and PUR show relatively higher levels of European, and to a lesser extent African, admixture than seen for PEL and MXL (Fig. 3d). We also attempted to infer Native American ancestry in admixed American populations by imputing polyTE genotypes for Native American populations from the Human Genome Diversity Project based on the 1000 Genome Project imputation panels. The ancestry contribution fractions for admixed American individuals are highly correlated between the observed Asian polyTE genotypes and the imputed Native American polyTE genotypes (Additional file 1: Figure S2).

Results of the same analyses are shown for individual polyTEs families in Additional file 1: Figures S3–S5. While the results are highly concordant for all three polyTE families, Alu ployTEs show the highest levels of resolution for human evolutionary relationships owing to the far higher number of polymorphic Alu insertions available for analysis. Nevertheless, L1 and SVA elements also show the ability to differentiate human populations and continental groups suggesting that these previously under-utilized polyTEs may also serve as useful ancestry markers.

### Ancestry prediction with polyTEs

Having established the overall ability of polyTE-based genotype analysis to capture known evolutionary relationships among human populations, we evaluated the ability of individual of polyTE loci to serve as useful markers for ancestry inference. To do this, levels of population differentiation for individual polyTE loci were assessed using the fixation index F_ST_ and the absolute allele frequency differences δ (see Methods). PolyTE loci-based F_ST_ and δ distributions were computed for three-way comparisons between non-admixed continental groups (African, Asian and European) and for five-way comparisons between individual populations within the same non-admixed continental group (Additional file 1: Figures S6 and S7). As can be expected, individual polyTE loci show substantially higher levels of population differentiation (i.e. higher F_ST_ and δ values) for the between compared to the within continental group comparisons. This is consistent with the overall ability of polyTE genotypes to better distinguish between continental groups (Fig. 3) than within continental groups (Additional file 1: Figure S8). The same pattern has been observed for SNP-based AIMs [36]. Nevertheless, polyTE loci are able to provide some level of resolution for even closely related populations within continental groups. A comprehensive list of human polyTE loci along with their allele frequencies and F_ST_ and δ values, within and between populations, are provided in Additional file 2: Table S1 so that investigators can choose loci of interest as potential ancestry markers.

Interestingly, the overall levels of polyTE-based F_ST_ are fairly low even for the between continental group comparison (Additional file 1: Figure S6). F_ST_ levels ≥ 0.4 have previously been taken to indicate that a nucleotide SNP can serve as a useful ancestry informative marker (AIM) [36, 37]. There are no individual polyTE loci that conform to this AIM criteria; 0.39 is the highest polyTE F_ST_ value. This can be attributed to the overall low frequency of polymorphic TE insertions seen here (Fig. 1a) since low levels of within-group polyTE allele frequency will depress F_ST_ levels owing to high levels of within group heterozygosity. The values of δ appear to be somewhat more sensitive for the characterization of individual polyTE AIMs. Several different δ value thresholds have been proposed for AIM characterization over the years [36]: 0.3, 0.4 and 0.5. There are 371 (0.3), 79 (0.4) and 9 (0.5) polyTE loci with continental δ values that exceed these thresholds. Thus, individual polyTE loci appear to have moderate ability to differentiate human populations, whereas ensembles of polyTE loci can be used effectively to distinguish more closely and distantly related populations.

**Fig. 4:**
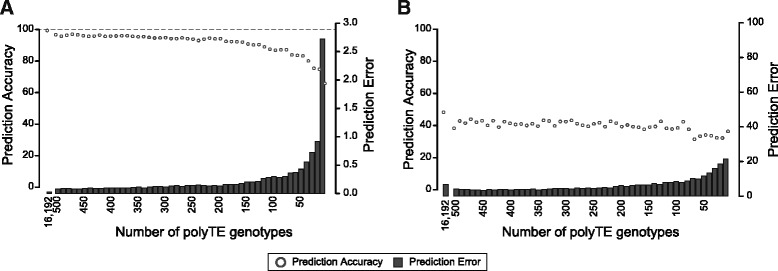
Ancestry predictions using polyTE genotypes. Relationship between the number of polyTE loci used to genotype individuals and the ancestry prediction accuracy for (**a**) continental population group comparisons (between African, Asian and European) and (**b**) sub-continental population comparisons (European)

In light of the ability of individual polyTEs genotypes and overall polyTE genotype patterns to differentiate human populations, we attempted to identify the smallest set of polyTE loci needed to accurately predict human ancestry. The accuracy of ancestry prediction was assessed for both non-admixed continental groups (African, Asian and European) and for individual populations within the African continental group. To do this for each comparison, the top 500 ancestry informative polyTE loci were ranked according to their F_ST_ levels and prediction accuracy was computed for sets of polyTE loci of sequentially decreasing size, going from 500 to 10 in steps of 10 (Fig. 4). Two measures of ancestry prediction, accuracy and error, were measured for each set of polyTE loci using the approach described in the Materials and Methods. When all polyTE loci are used, continental group ancestry prediction approaches 100 % accuracy with < 1 % error. As the number of polyTE loci used for ancestry prediction is steadily decreased from 500, the accuracy declines and the error increases. However, the changes in accuracy and error are relatively slight. For the top 100 polyTE loci, ancestry prediction is 86.9 % accurate with 0.3 % error. The smallest set of 10 polyTE loci yields 65.8 % accuracy and 2.7 % error. These results are similar to a previous report [27] that evaluated the minimum number of polymorphic Alu loci (~50) that would yield accurate genetic distances between human populations.

A similar approach was taken to evaluate the utility of polyTE genotypes for ancestry prediction within continental groups. Consistent with what is observed for the within continental group F_ST_ values (Additional file 1: Figure S6), polyTE genotypes have less power to discriminate ancestry for closely related populations from the same continental group (Fig. 4b). For the African populations, individual genotypes based on the entire set of polyTE loci yield an ancestry prediction accuracy of 48.3 % and an error of 6.7 %. Since there are five African populations, a random predictor would yield 20 % accuracy. Thus, the accuracy achieved by polyTE loci, while relatively low, is 2.4x greater than expected by chance alone. Accuracy does not change greatly with decreasing numbers of polyTE loci. 100 polyTE loci yields accuracy of 38.5 %, and the accuracy for 10 polyTE loci is 36.3 %. The error rate of prediction does steadily increase to 8.4 % for 100 polyTE loci and 21.3 % for 10 polyTE loci.

### Admixture prediction with polyTEs

**Fig. 5:**
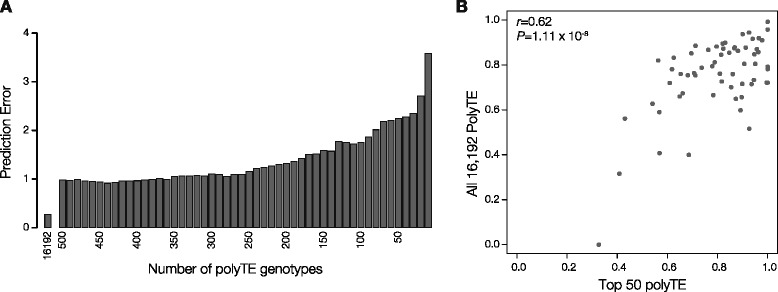
Admixture predictions using polyTE genotypes. **a**. Relationship between the number of polyTE loci used to genotype individuals and admixture prediction accuracy for the ASW population. **b**. Comparison of individual admixture proportions calculated using all available polyTE genotypes versus a minimal polyTE genotype set with 50 loci

Having established the utility of small sets of polyTE loci to make ancestry inferences for non-admixed groups, we wished to similarly evaluate the ability of polyTE loci sets to allow for inferences about continental ancestry contributions to admixed populations. To do this, ancestral contributions from African and European populations to the admixed ASW American population were evaluated using sets of polyTE loci of decreasing size in a similar way as was done for ancestry prediction in non-admixed populations. In the case of admixture, prediction error levels were measured by comparing the ancestral admixture components computed from the entire set of 16,192 polyTE loci to those computed from the smaller polyTE loci sets (see Methods). As with ancestry prediction, error levels steadily increase with the use of decreasing numbers of polyTE loci (Fig. 5a). However, slightly larger numbers of polyTE loci are required to keep admixture inference error levels low; the use of 10 polyTE loci yields 3.4 % error, whereas a set of 50 polyTE loci reduces the error to 2.2 %. There is strong agreement in the results of continental ancestry contributions for this admixed population between analyses conducted with all polyTEs versus the top 50 polyTEs (*r* = 0.62; Fig. 5b).

## Discussion

### Human ancestry and admixture from polyTEs

Our analysis of a genome-wide set of human polyTE genotypes indicates that TE polymorphism patterns recapitulate the pattern of human evolution and migration over the last 60–100,000 years (Fig. 3 and Additional file 1: Figures S3–S5). While polyTEs considered as an ensemble provide substantial resolution for inferring ancestry and human relationships, individual polyTE loci show moderate population differentiation levels (Additional file 1: Figure S6 and S7). This can be attributed to the fact that individual polyTE loci tend to be found at low allele frequencies (Fig. 1a). However, these same low frequency loci do show high levels of geographic differentiation, i.e. many of them are continental group or population specific (Fig. 1b). Therefore, when a relatively small set of these low frequency but highly geographically differentiated polyTE loci are used together, they do in fact provide substantial resolution for evolutionary analysis as well as ancestry and admixture inference (Figs. 4 and 5).

These results have important implications for the study of human evolution, ancestry and admixture by smaller labs that may not have access to the same level of resources as larger consortia or genome centers since analysis of a small set of polyTE loci (10–50 depending on the application) can prove to be quite informative. Given the size range of TEs insertions, in particular for Alus which are the most numerous family of polyTEs, element presence/absence patterns can be accurately characterized in a cost-effective way using (multiplex) PCR-based techniques. Protocols for PCR-based analysis of polyTEs are well established in a number of labs. The results of this study can be used to help investigators choose the specific TE loci of interest for their own evolutionary studies (see Additional file 2: Table S1 for a list of genomic locations of polyTEs and their allele frequencies and F_ST_ values).

Despite the overall utility of polyTEs as ancestry markers, results from this study suggest that they are not likely to be good markers for mapping by admixture linkage disequilibrium (MALD or admixture mapping) studies [38, 39]. These studies rely on detailed locus-specific assignments of ancestry across the genome in admixed individuals. In order to achieve this level of resolution, thousands of markers are needed and individual markers should have high levels of population differentiation (as measured by F_ST_ or other related metrics) [36]. Thus, SNPs would seem to remain the best choice of AIMs for MALD (admixture mapping) studies.

### Deleteriousness and selection on polyTE insertions

Our initial analysis of human polyTEs within and between populations revealed that TE insertion polymorphisms are found at very low frequencies (Fig. 1a). This is consistent with the overall deleteriousness of TE insertions and accordingly their removal by purifying selection. The elimination of polyTEs by purifying selection is also underscored by the fact that polyTEs are vastly under-represented in genic and exonic regions (Additional file 1: Figure S9). Nevertheless, some polyTEs do rise to high allele frequencies and many also show high levels of geographic differentiation consistent with what has been seen for SNPs [28]. This differentiation is precisely what makes them good markers for ancestry inference, particularly when considered as an ensemble, but it also suggests the possibility that polyTE insertions may influence population specific phenotypes shaped by selection. Additional analysis on the effects of selection on TE polymorphisms, as well as the relationship between polymorphic TEs and potentially adaptive phenotypes, will be needed to test this assertion.

## Conclusions

Polymorphic TE loci have long been used as markers in human population genetic studies, and they are known to provide a number of advantages for such studies. The selection of which polyTE loci to use for population genetic studies has been largely *ad hoc*, based on a combination of literature and database surveys together with empirical evaluation on the suitability of individual loci as markers that can discriminate between populations. With the recent release of a genome-wide set of 16,192 TE polymorphisms by the 1000 Genomes Project [28, 35], genotyped across 2,504 individuals from 26 global populations, it is now possible to systematically evaluate the utility of polyTE loci for human population genetic and ancestry studies. We have leveraged these newly released data to conduct the first genome-scale analysis of polyTE genotypes for the study of human genetic ancestry. We show that the genetic diversity represented by TE polymorphisms reflects known patterns of human evolution, and define sub-sets of polyTE loci that can be used as ancestry informative markers. We provide ranked lists of the polyTE loci that can be used by researchers in the community for future ancestry and admixture analyses.

## Methods

### Transposable element polymorphisms

Human polymorphic transposable element (polyTE) genotypes were taken from the Phase3 data release (November 2014) of the 1000 Genomes Project [28, 35] (ftp://ftp-trace.ncbi.nih.gov/1000genomes/ftp/release/20130502/). These genotypes consist of phased presence/absence patterns of polyTE insertions at specific human genome sites for individual genomes, and they are characterized from human genome reference sequence mapped next-generation sequence data via 1) discordant read mapping for short paired-end reads and/or 2) split read mapping for longer reads as previously described [40]. PolyTE allele frequencies are calculated as the number of present TE insertions (*TEi*) normalized by the total number of sites in the population (*2n*): *TEi/2n*. The extent to which individual polyTE loci differentiate populations was computed using the fixation index F_*ST*_ with the Weir Cockerham method [41] implemented in VCFtools [42] and the δ parameter [36], which is defined as the absolute value of the difference in the allele frequencies between populations for TE polymorphisms.

### Ancestry analysis

PolyTE-based allele sharing distances were computed for all pairs of human genomes by counting the total number of polyTE presence/absence alleles that differ between two individuals across all genomic insertion sites. Allele sharing distances computed in this way were projected in two-dimensional space using multi-dimensional scaling (MDS) implemented in R. This was done for pairwise distances computed between individual genomes and for average allele sharing distances among populations. Population average allele sharing distances were used to reconstruct a neighbor-joining [43] phylogenetic tree using the program MEGA6 [44].

### Admixture analysis

The program ADMIXTURE was used to infer the proportion of ancestry contributions from ancestral populations to modern admixed populations from the Americas (ACB, ASW, CLM, MXL, PEL, PUR) based on polyTE genotypes. The program was first run in supervised mode with three ancestral clusters: African, Asian and European. Asian ancestry is taken here as a rough surrogate for Native American admixture in American populations given the relatively close evolutionary relationship between East Asian and Native American populations and the lack of Native American samples in the 1000 Genomes Project. PolyTE genotypes were then imputed for Native American genomes from the Human Genome Diversity Project [31, 45], using the impute panel from the 1000 Genomes Project with the program IMPUTE2 [46], and ADMIXTURE was run in supervised mode with the three ancestral clusters: "African, European and Native American. The ancestry contribution fractions for modern admixed populations from the Americas computed based on observed Asian polyTE genotypes and imputed Native American genotypes were correlated to check for consistency.

### Ancestry and admixture prediction analyses

The program ADMIXTURE was used together with a cross-validation approach in order to predict the ancestry of individuals based on their polyTE genotypes. The cross-validation method relied on an 80 %/20 % split of the data, whereby 80 % of individual polyTE genotypes were used to build a three-cluster ancestry model with ADMIXTURE. The remaining 20 % of individual polyTE genotypes were then tested against this model to predict their ancestry membership in one of the three groups. Group-specific ancestry was only assigned if the probability of group membership was calculated as ≥ 90 %. Accuracy is then defined as the number of correct ancestry predictions normalized by the total number of predictions made. Error is defined as the root-mean-square difference (*RMSD*) between the predicted and actual ancestry inference made with the complete data. *RMSD* values are reported as the average prediction error for all individuals. This process was done repeatedly across individual polyTE genotypes based on decreasing numbers of polyTE sites, from 500 to 10 in steps of 10. For each polyTE set, this 80/20 prediction process was repeated 100 times.

An analogous prediction approach was used to infer the continental ancestry contributions to an admixed American population (ASW) using ADMIXTURE. In this case, the training was done using individual polyTE genotypes from ancestral populations (African and European) and the testing was done using polyTE genotypes from admixed ASW individuals. This was done first using all 16,192 polyTE loci and then for individual polyTE genotypes based on decreasing numbers of polyTE sites, from 500 to 10 in steps of 10. The predicted ancestry contributions to admixed individuals were compared for results based on all polyTE loci and results based on reduced sets of polyTE loci using the root-mean-square difference (*RMSD*) for the African and European fractional ancestry contributions.

## Additional files

Additional file 1:
**Contains Figures S1–S9 and figure legends.** (PDF 5864 kb) Additional file 2: Table S1. List of human polyTE loci with allele frequencies and F_ST_ and δ values. (XLSX 73 kb)

## Abbreviations

TE: Transposable Elements
polyTE: Polymorphic Transposable Elements
LINE: Long Interspersed Nuclear Elements
SINE: Short Interspersed Nuclear Elements
SNP: Single Nucleotide Polymorphism
AIM: Ancestry Informative Marker
MDS: Multi-Dimensional Scaling
MALD: Mapping by Admixture Linkage Disequilibrium
RMSD: Root-Mean-Square Difference
