# Supplementary material for: Transposable element polymorphisms recapitulate human evolution
Source: Mob DNA. 2015 Nov 16;6:21. doi: 10.1186/s13100-015-0052-6 (PMC4647816; doi:10.1186/s13100-015-0052-6)
Supplement: Additional file 1: — Contains Figures S1–S9 and figure legends. (PDF 5864 kb) [file 13100_2015_52_MOESM1_ESM.pdf]

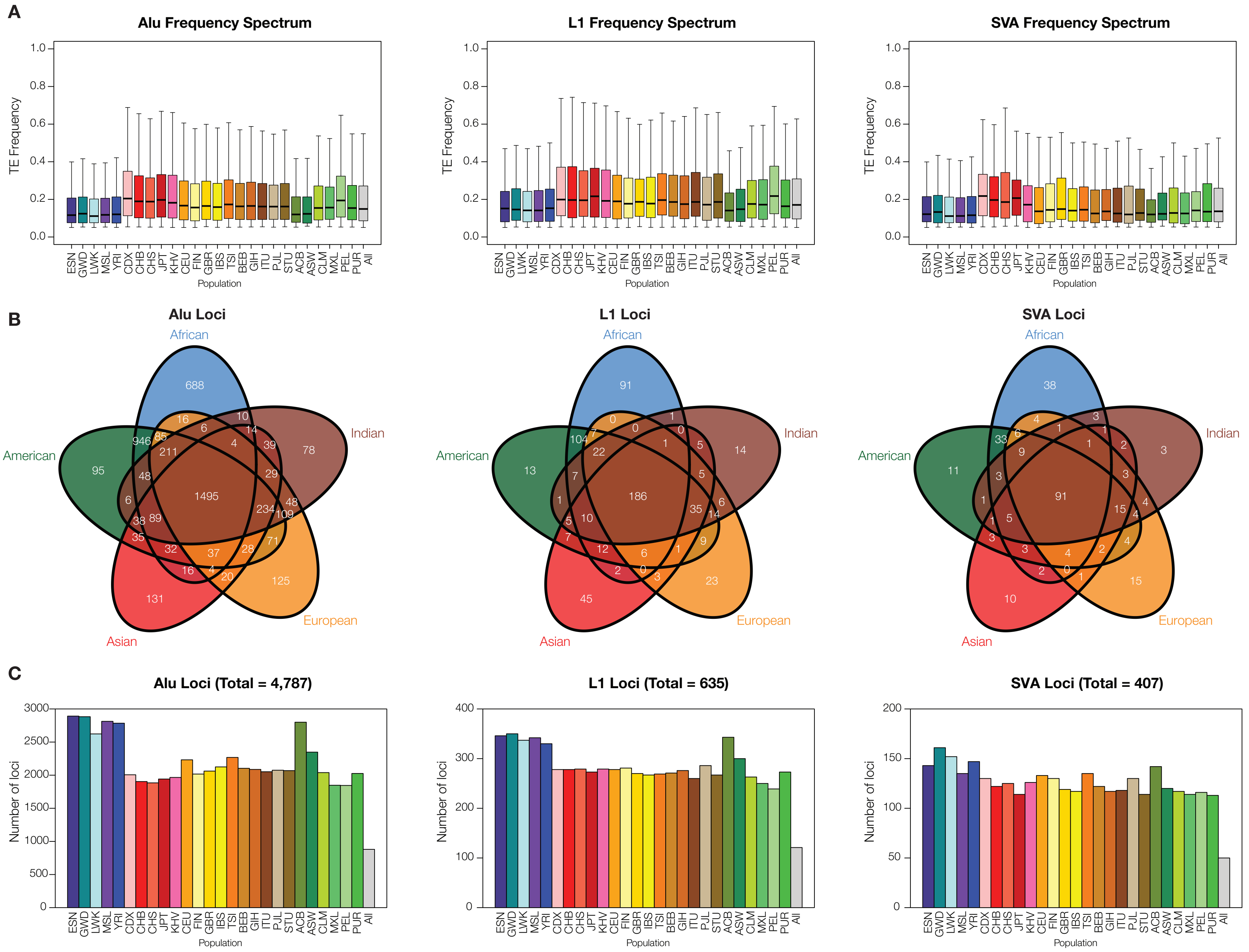

Figure S1. **Distribution of polymorphic transposable element (polyTE) loci among human populations**. Data are shown individually for Alu, L1 and SVA polyTEs. Populations are organized into five continental groups (see Table 1): African (blue), Asian (red), European (gold), Indian (brown) and American (green). (A) polyTE allele frequency distribution. (B) Number of polyTE loci. (C) Numbers of shared and exclusive polyTE loci.

A

Population	Number of Samples
**Brazil**	**45**
Karitiana	24
Surui	21
**Colombia**	**13**
Colombians	13
**Mexico**	**50**
Maya	25
Pima	25
**Total**	**108**

D

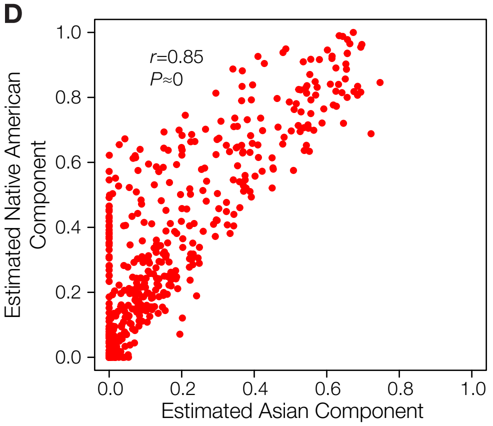

B

C

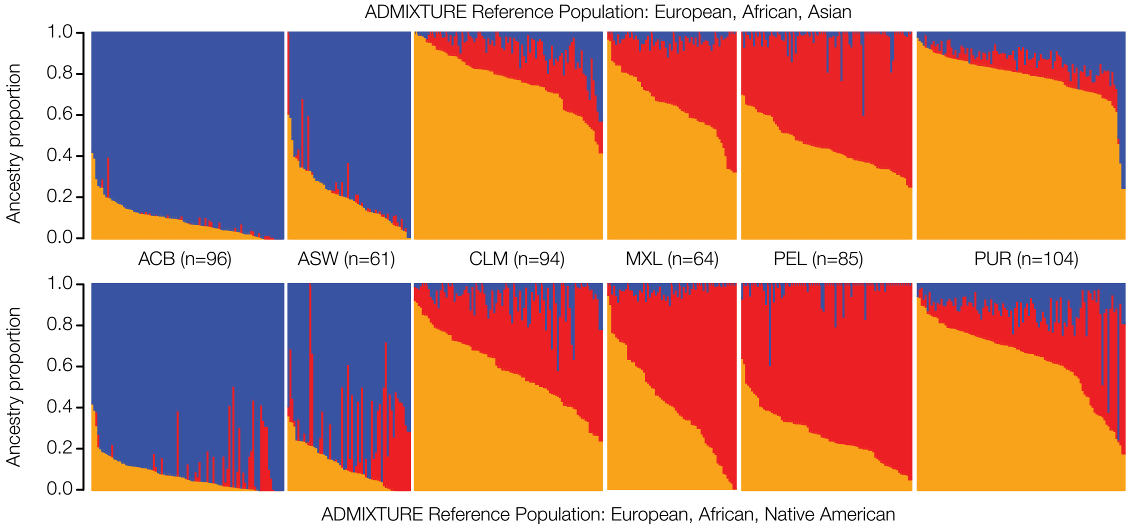

Figure S2. **Continental ancestry contributions for individuals from admixed populations computed using observed Asian versus imputed Native American polyTE genotypes.** (A) Native American populations used for polyTE genotype imputation. Ancestry contribution fractions for admixed individuals computed using (B) Asian polyTE genotypes as a surrogate for Native American ancestry and (C) polyTE genotypes imputed for Native American populations. (D) Correlation between the ancestry contribution fractions computed using Asian versus Native American polyTE genotypes.

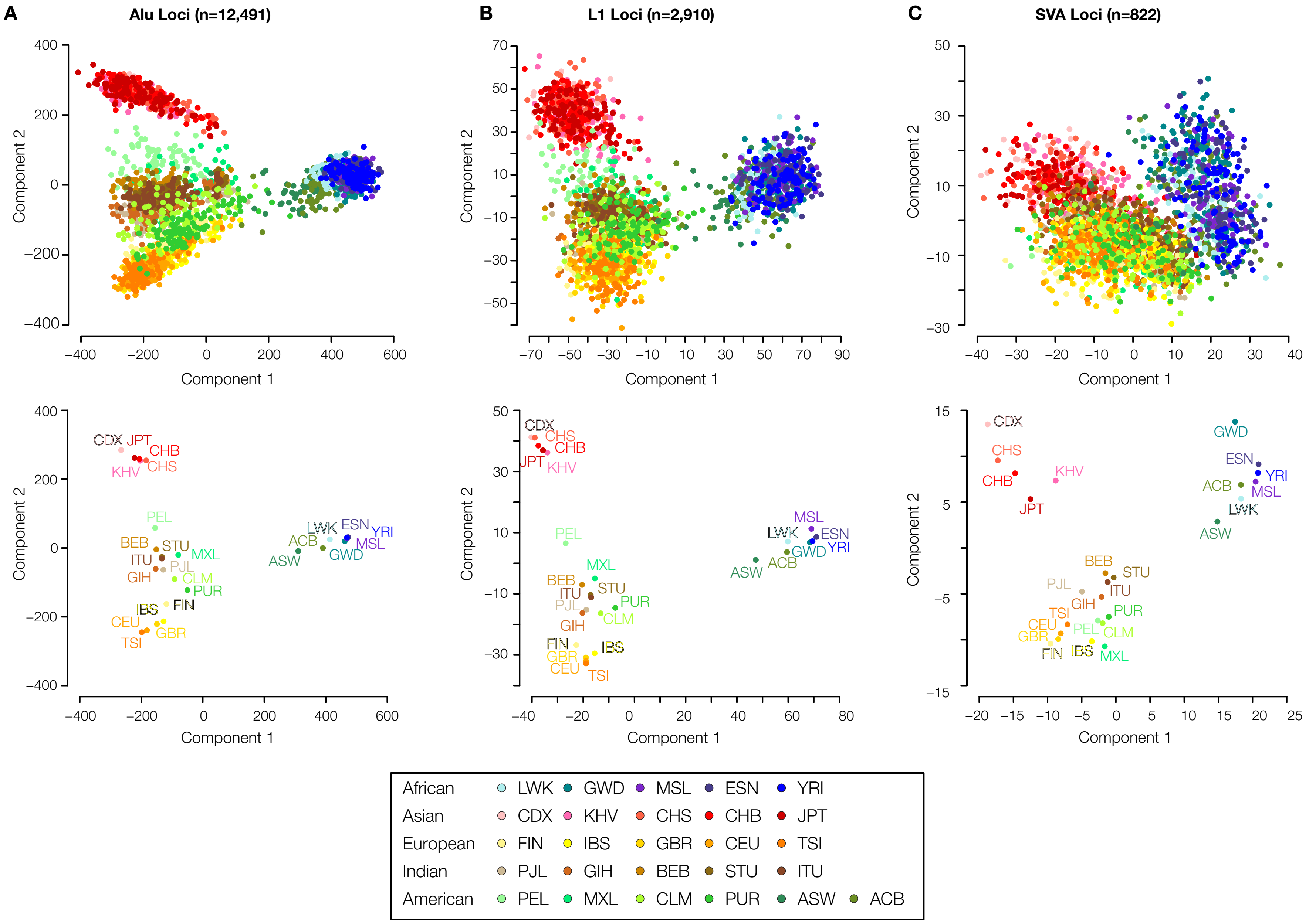

Figure S3. **Clustering of human populations based on polyTE genotypes. Populations are color coded as shown in the figure legend**. Multi-dimensional scaling (MDS) plots showing polyTE genotype-based relationships among 2,504 individuals from 26 human populations are shown for (A) polyAlu, (B) polyL1 and (C) polySVA loci. The bottom panels show the same polyTE genotype MDS plots based on population average relationships.

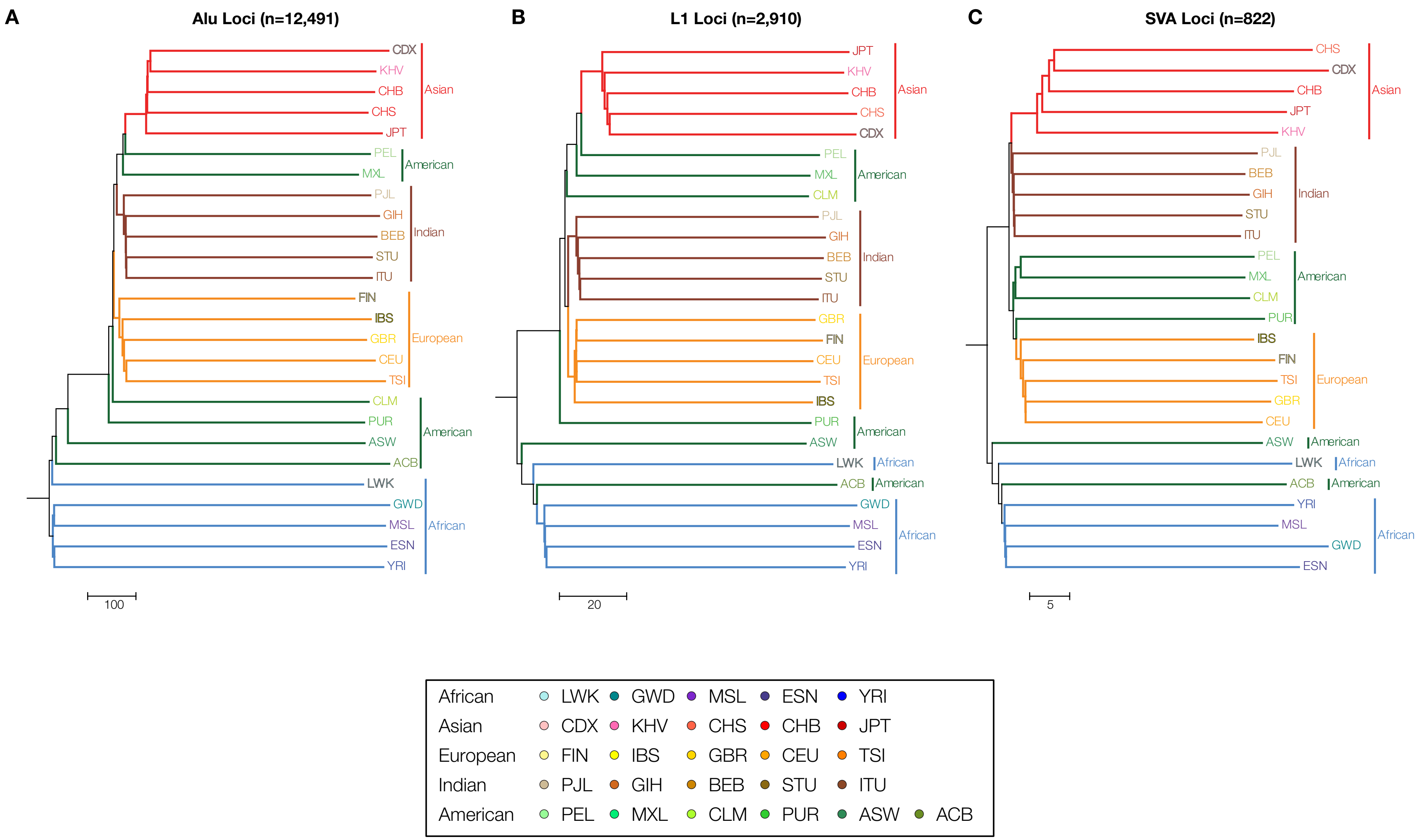

Figure S4. **Phylogenetic relationships among human populations based on polyTE genotypes**. Populations are color coded as shown in the figure legend. Phylogenetic trees based on average polyTE allele sharing distances between human populations are shown for (A) polyAlu, (B) polyL1 and (C) polySVA loci.

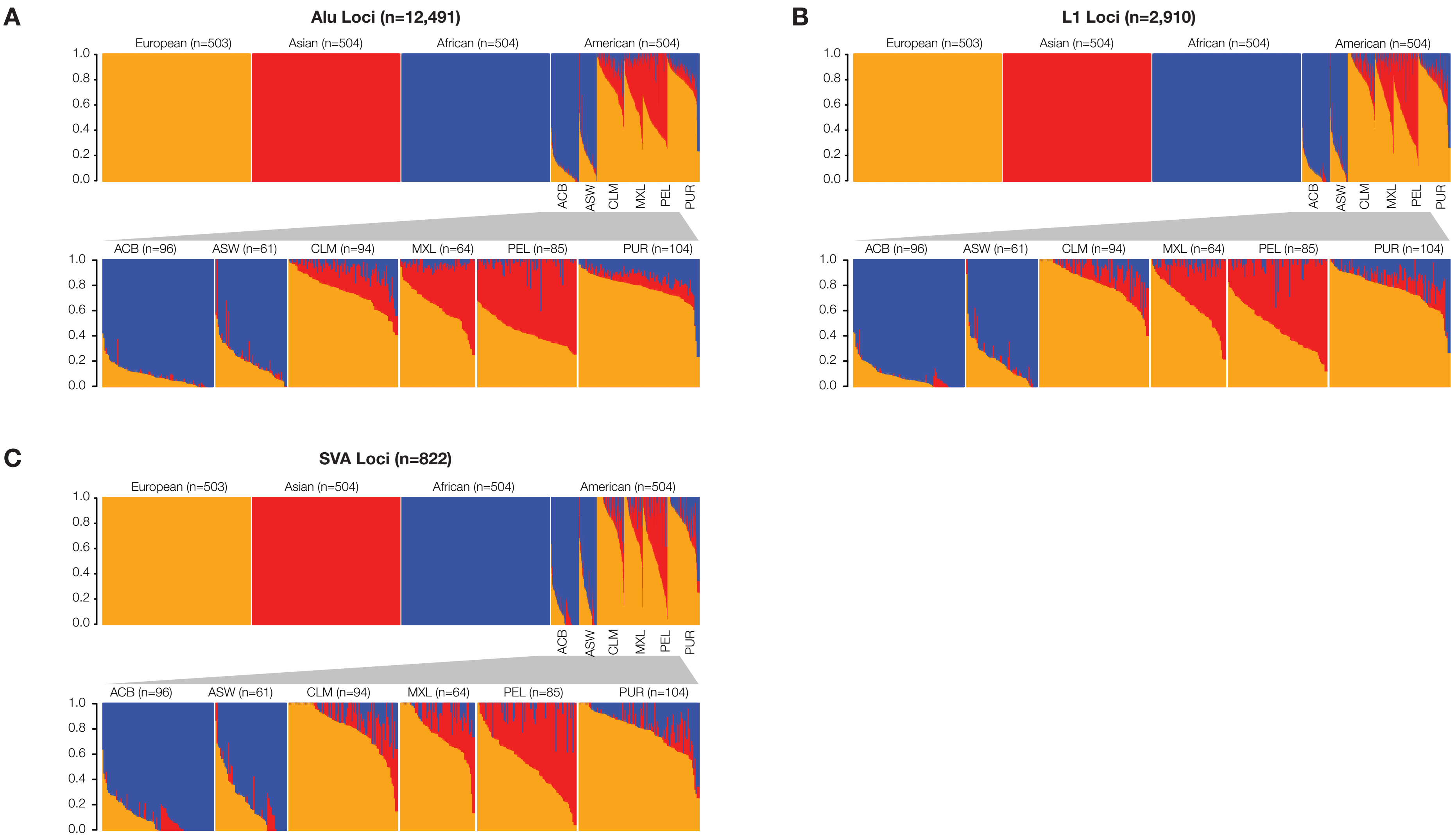

Figure S5. **Continental ancestry contributions for individuals from ancestral and admixed human populations**. polyTE genotype-based continental ancestry contribution fractions for individuals from non-admixed ancestral (European, Asian and African) and admixed (American) human populations are shown for (A) polyAlu, (B) polyL1 and (C) polySVA loci.

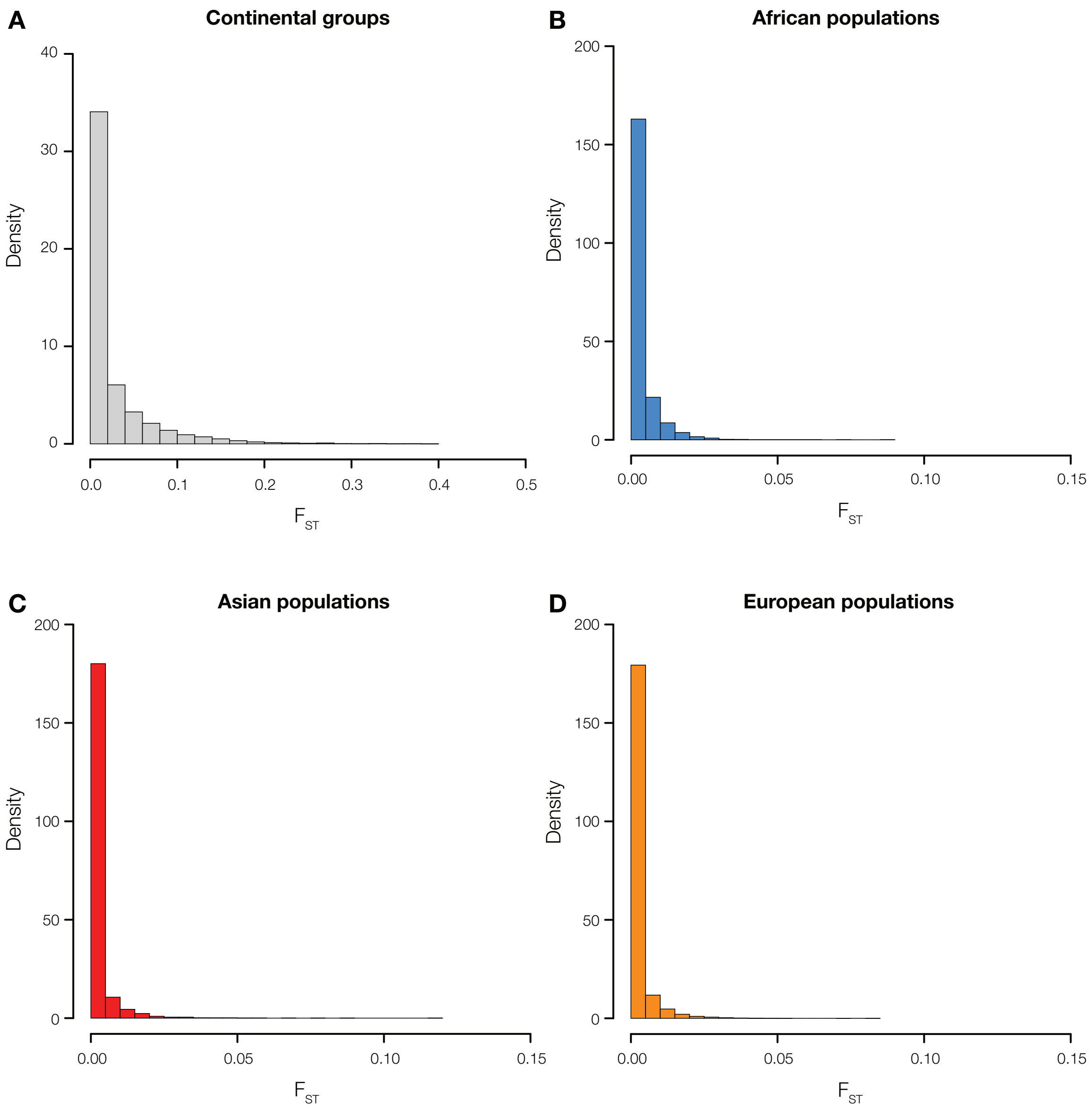

Figure S6. **PolyTE genotype F_{ST} value distributions for continental group and subcontinental population comparisons**. Density distributions of F_{ST} values for polyTE genotypes are shown for (A) between continental group comparisons (African, Asian and European) and for sub-continental comparisons between (B) African, (C) Asian and (D) European populations.

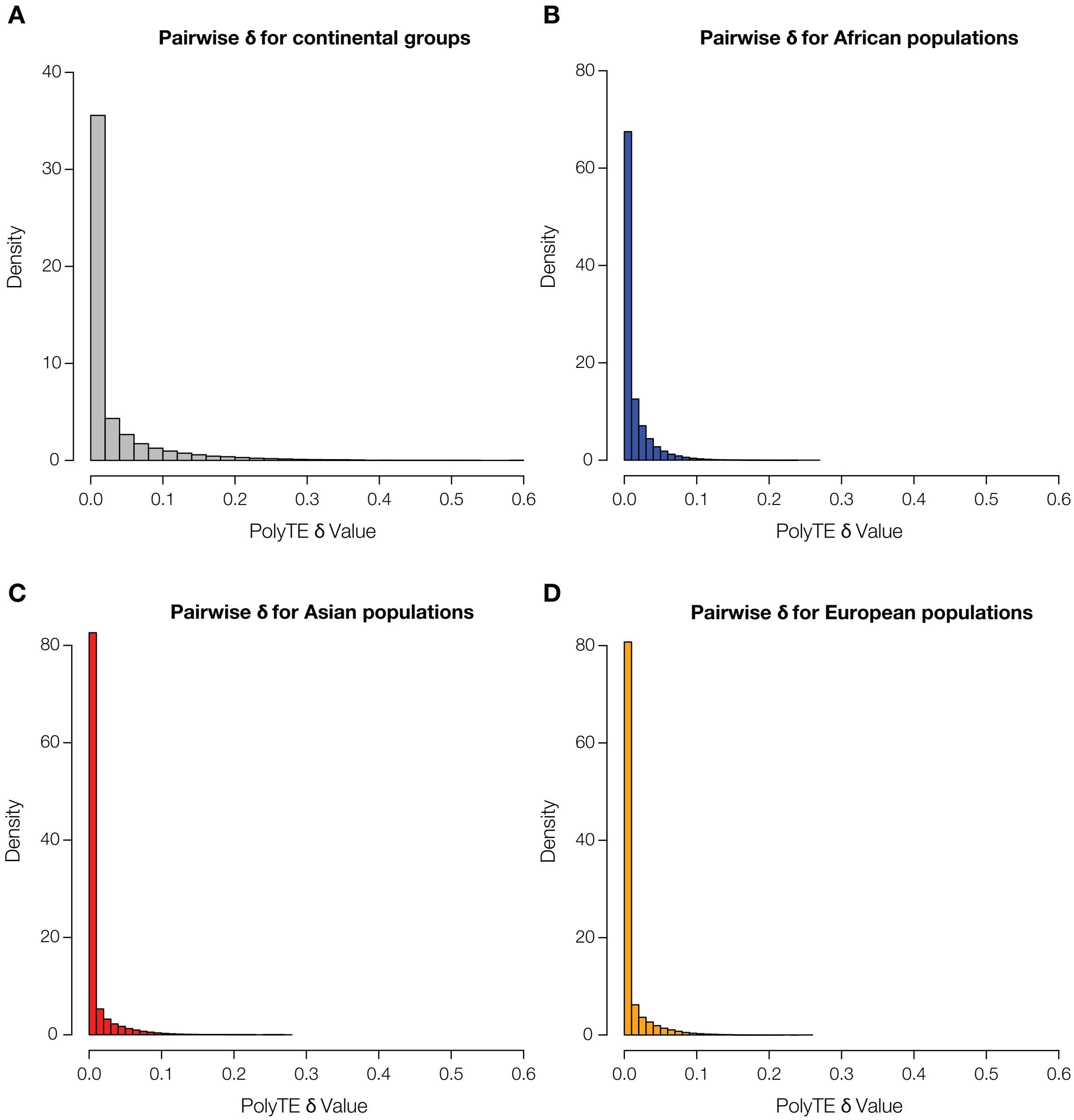

Figure S7. **PolyTE genotype pairwise δ value distributions for continental groups and subcontinental population comparisons**. Density distributions of all pairwise δ values for polyTE genotypes are shown for (A) between continental group comparisons (African, Asian and European) and for sub-continental comparisons between (B) African, (C) Asian and (D) European populations.

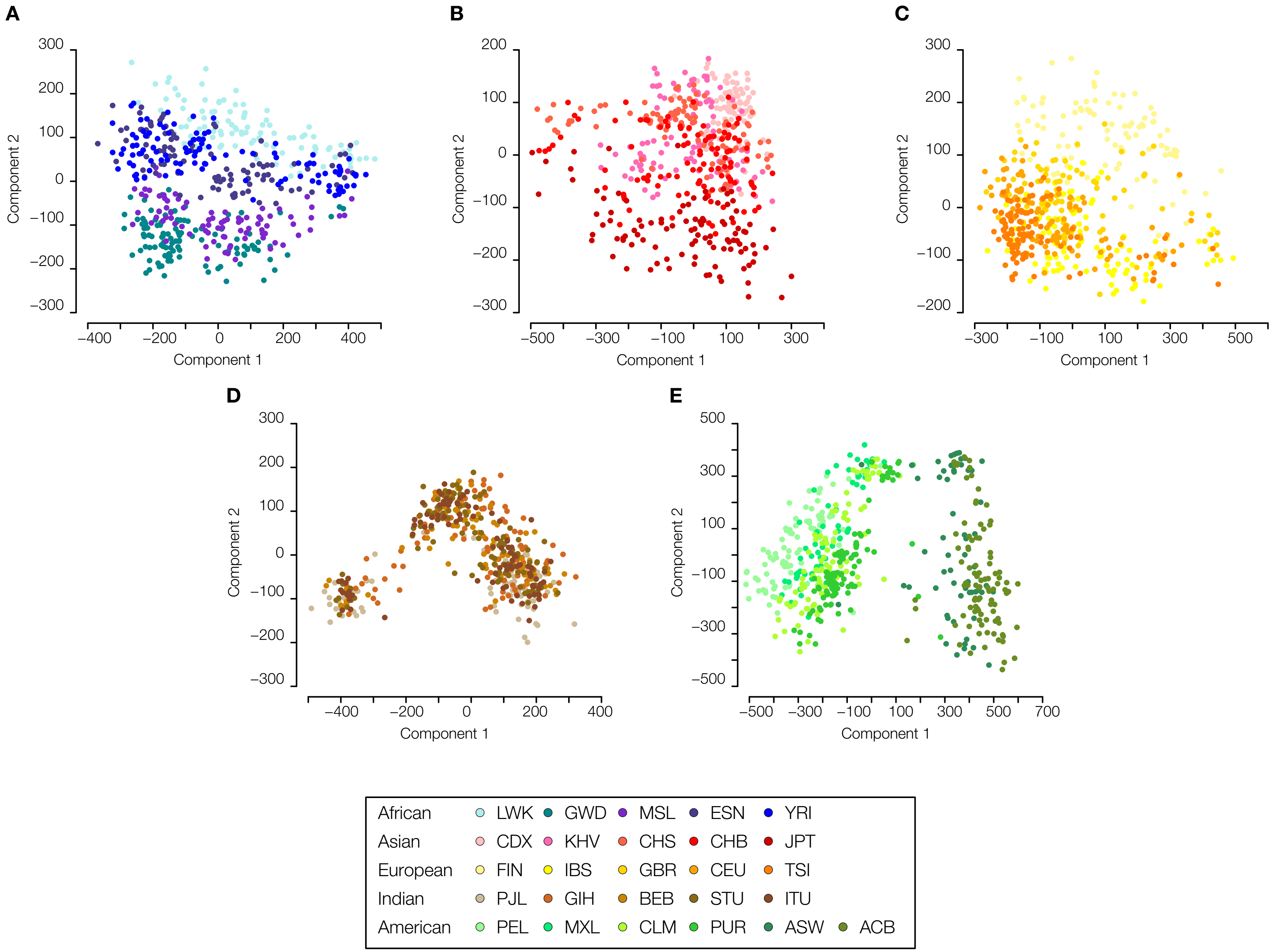

Figure S8. **Sub-continental evolutionary relationships among human populations base on polyTE genotypes**. Populations are color coded as shown in the figure legend. Multi-dimensional scaling (MDS) plots showing polyTE genotype-based relationships among (A) African, (B) Asian, (C) European, (D) Indian and (E) American populations.

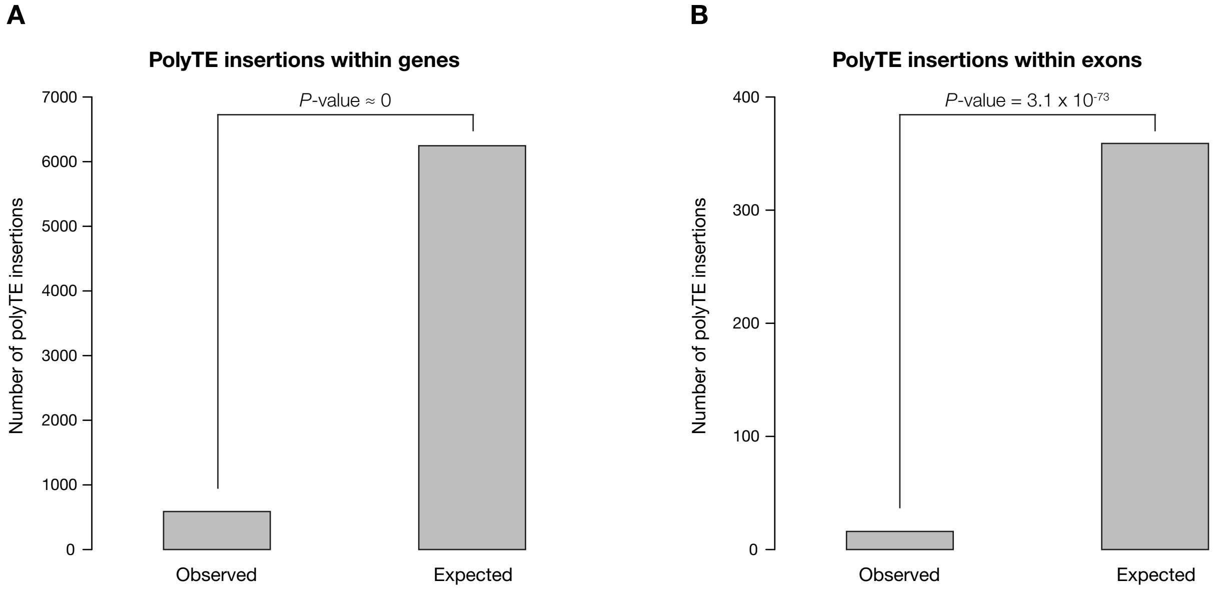

Figure S9. **Numbers of polyTE insertions found within genes and exons**. The observed numbers of polyTE insertions are compared to the expected numbers for (A) gene regions and (B) exons. The expected numbers are computed based in the total number of polyTE insertions genome-wide and the fraction of the genome made up by genes (taken from Refeq transcription start to termination sites) and exons (taken from Refseq exon start to end sites). *P*-values are based on χ^2 tests.
